# Quantitative analysis of proteostasis disruption following siRNA-mediated HSP70 depletion in HeLa cells

**DOI:** 10.3389/fgene.2026.1856671

**Published:** 2026-09-28

**Authors:** Hatice Esenkaya

**Affiliations:** 1 Department of Cell and Molecular Biology, Karolinska Institutet, Stockholm, Sweden; 2 School of Life Sciences, Molecular Biology and Genetics, Kilis 7 Aralik University, Kilis, Türkiye

**Keywords:** Ras GTPase-activing protein-binding protein 1, HeLa cells, heat shock protein 70, molecular chaperones, protein aggregation, proteostasis, small interfering RNA

## Abstract

**Introduction:**

Heat shock protein 70 (HSP70) is a central molecular chaperone that maintains proteostasis by supporting protein folding, preventing protein aggregation, and coordinating cellular stress responses. Dysregulation of HSP70 has been associated with cancer progression, neurodegenerative disorders, and aging-related proteotoxicity. However, many experimental approaches rely on chemical inhibition or stress-inducing conditions that may introduce additional confounding effects. This study aimed to investigate the proteostatic consequences of HSP70 depletion in the absence of stress-inducing chemical inhibitors.

**Methods:**

A rapid, quantitative workflow was developed to assess chaperone function and proteostasis in cultured cells. HSP70 was depleted in HeLa cells using siRNA-mediated gene silencing. Knockdown efficiency was evaluated at the transcript and protein levels using quantitative polymerase chain reaction (qPCR) and Western blotting, respectively. Flow cytometry was used to quantify ProteoStat-detected misfolded/aggregated protein burden and total cellular fluorescence from a G3BP1-GFP reporter.

**Results:**

siRNA-mediated HSP70 depletion resulted in a greater than 90% reduction in HSP70 transcript and protein levels. HSP70-depleted cells exhibited a 1.8-fold increase in ProteoStat fluorescence, indicating an increased burden of misfolded/aggregated proteins, and a 2.9-fold increase in total cellular G3BP1-GFP fluorescence. Because flow cytometry does not resolve intracellular localization or puncta formation, the increased G3BP1-GFP fluorescence should be interpreted as a surrogate reporter response and does not constitute direct evidence of stress granule biogenesis.

**Discussion:**

These findings are consistent with impaired proteostasis following acute HSP70 depletion and demonstrate the potential utility of an inhibitor-independent workflow for evaluating cellular responses associated with targeted chaperone depletion. Orthogonal spatial validation using immunofluorescence or confocal microscopy is required to determine G3BP1 localization and confirm stress granule formation. In addition, experiments using multiple independent siRNA duplexes will be necessary to establish target specificity and strengthen the mechanistic interpretation of the observed proteostatic response.

## 1 Introduction

Protein homeostasis (proteostasis) is essential for cellular survival, encompassing processes that ensure proteins are correctly folded, functional, and appropriately degraded when damaged (Ottens et al., 2021; Hipp and Hartl, 2024). Perturbation of the proteostasis network is strongly associated with diseases (Lim and Vendruscolo, 2025), such as cancer (Wei et al., 2024), amyotrophic lateral sclerosis (ALS) (Tseng et al., 2023), and Alzheimer’s disease (Cozachenco et al., 2023), and proteostasis deteriorates with aging (Kedersha et al., 2005). Therefore, there are certainly therapeutic opportunities toward managing proteostasis (Kuzu et al., 2025). Central to this system are the heat shock proteins (HSPs), a family of highly conserved molecular chaperones that buffer cells against proteotoxic stress (Mayer and Bukau, 2005; Keller, 2025; Alagar Boopathy et al., 2022). Among them, heat shock protein 70 (HSP70) plays a pivotal role by stabilizing nascent polypeptides, preventing protein aggregation, promoting refolding of misfolded proteins, and cooperating with ubiquitin–proteasome and autophagy pathways (Singh et al., 2025).

Recent studies have highlighted an expanded view of HSP70 function beyond canonical chaperoning, with HSP70 acting as a key regulator of messenger ribonucleoprotein (mRNP) remodeling (Kedersha et al., 2005; Kishor et al., 2017), ncRNA biogenesis (Leone et al., 2024), and cytoplasmic stress granule dynamics (Jain et al., 2016; Ganassi et al., 2016). Stress granules are dynamic membrane-less condensates containing untranslated mRNAs, stalled translation initiation complexes, and RNA-binding proteins, including Ras GTPase-activating protein-binding protein 1 (G3BP1), which serves as a core nucleating factor (Adjibade and Mazroui, 2023; Yang et al., 2020; Smith and Bartel, 2025). Under conditions of oxidative stress, heat shock, or proteotoxic insults, HSP70 helps maintain the solubility of stress granule components and participates in granule disassembly once stress is resolved (Ganassi et al., 2016; Hu et al., 2022). Disruption of HSP70 and other HSP activities has been shown to enhance stress granule persistence (Ma et al., 2017; Wang et al., 2022), increase the load of misfolded proteins, and predispose cells to pathological protein aggregation, which is a hallmark of several neurodegenerative diseases (Park et al., 2018; Sharma et al., 2025; Younas and Zerr, 2025). However, much earlier work relies on chemical inhibition or extreme stress induction, which can activate secondary stress pathways (Hu et al., 2022) or cause off-target effects, complicating interpretation of HSP70’s direct role in stress granule homeostasis (Schlecht et al., 2013; Du et al., 2022). In this study, we establish a rapid, inhibitor-independent multi-assay workflow to quantitatively assess proteostasis after acute siRNA-mediated depletion of HSP70. Rather than establishing a previously unknown biological role of HSP70, the primary contribution of the present study is the development and validation of an integrated, inhibitor-independent quantitative workflow that combines transcript-level, protein-level, and functional proteostasis measurements within a single experimental pipeline. This workflow provides a reproducible framework that can be readily adapted for studying additional molecular chaperones under genetically defined conditions.

## 2 Materials & methods

### 2.1 Cell culture

HeLa human cervical carcinoma cells (ATCC CCL-2, 10,801 University Boulevard, Manassas, VA 20110–2209, United States) were used for all experiments and maintained according to standard cell culture procedures. In brief, cells were revived from low-passage frozen stocks and cultured in high-glucose Dulbecco’s modified Eagle medium (DMEM; Thermo Fisher Scientific, Cat. 11965092) supplemented with 10% heat-inactivated fetal bovine serum (FBS; Thermo Fisher Scientific, Cat. number 26140079) and 1% penicillin–streptomycin (100 U/mL penicillin and 100 μg/mL streptomycin; Gibco, Cat. #15140122). Cultures were maintained at 37 °C in a humidified atmosphere containing 5% CO_2_ and passaged at approximately 70%–80% confluency by washing once with sterile phosphate-buffered saline (PBS) and detaching the monolayer with 0.25% trypsin–EDTA (Gibco, Cat. #25200056). The enzymatic reaction was quenched with complete medium, and cells were resuspended to obtain a single-cell suspension before reseeding at densities optimized for each downstream assay. For experiments, cells were plated 24 h prior to treatment to allow proper attachment and recovery, typically at densities of 2–3 × 10^5^ cells per well in 6-well plates, 1–1.5 × 10^5^ cells per well in 12-well plates, or 5–10 × 10^3^ cells per well in 96-well plates. Cells were authenticated by STR profiling as reported by the supplier. To ensure experimental reliability, medium color, pH, and cell morphology were checked daily, and cultures were confirmed negative for *mycoplasma* contamination during monthly screening using a PCR-based detection assay. Aseptic techniques were strictly followed during all procedures, and only cultures exhibiting normal epithelial morphology and exponential growth were used for analyses.

### 2.2 siRNA transfection

For gene-silencing experiments, HeLa cells were transfected with a single small interfering RNA (siRNA) duplex targeting HSP70. The specific siRNA sequences used were the sense strand 5′-GCA​AGA​UCU​UCU​CUA​CUA​Att-3′ and the antisense strand 5′-UUA​GUA​GAG​AAG​AUC​UUG​Ctt-3′. A non-targeting control siRNA (Dharmacon, Cat. #D-001810–01–05) was used in parallel experiments. Transfections were performed using Lipofectamine RNAiMAX reagent (Thermo Fisher Scientific, Cat. #13778150) according to the manufacturer’s recommendations. In brief, cells were seeded at a density of 2 × 10^5^ cells per well in 6-well plates 1 day prior to transfection to ensure optimal adherence. For each well, 50 nM siRNA was diluted in Opti-MEM reduced-serum medium (Thermo Fisher Scientific) and combined with the transfection reagent to form siRNA–lipid complexes, which were added directly to the cells. After a 6-h incubation period, the transfection mixture was replaced with complete growth medium, and cells were maintained for an additional 48 h before being harvested for downstream analyses. In preliminary optimization experiments performed under identical conditions, transfection efficiency was estimated to exceed 90% using a fluorescently labeled control siRNA.

### 2.3 qPCR

Reverse-transcriptase quantitative real-time PCR (RT-qPCR) was performed to evaluate HSP70 mRNA expression following siRNA-mediated knockdown. Total RNA was extracted from HeLa cells using the RNeasy Mini Kit (QIAGEN, Cat. #74106) according to the manufacturer’s protocol, and then RNA concentration and purity were assessed by spectrophotometry. For cDNA synthesis, 1 µg of total RNA was reverse-transcribed using the iScript cDNA Synthesis Kit (Bio-Rad, Cat. #1708891) in a 20 µL reaction volume. Quantitative PCR reactions were prepared using SYBR Green Master Mix (Thermo Fisher Scientific, Cat. #4367659) and gene-specific primers. The primer sequences used were as follows: HSP70 forward 5′-ATG​GCC​AAA​GCC​GCG​GCG-3′ and reverse 5′-TGA​CCT​CGA​GGT​CCT​TGA​G-3′; GAPDH forward 5′-GAA​GGT​GAA​GGT​CGG​AGT-3′ and reverse 5′-GAA​GAT​GGT​GAT​GGG​ATT​TC-3′. Reactions were run on a real-time PCR system using the following thermal cycling conditions: 95 °C for 2 min, followed by 40 cycles of 95 °C for 15 s and 60 °C for 30 s. Melt curve analysis was performed at the end of each run to confirm primer specificity. Relative mRNA expression levels were calculated using the 2^−ΔΔCt^ method, with GAPDH as the endogenous reference gene and the non-targeting siRNA group as the calibrator.

### 2.4 Western blot

Western blotting was performed to assess HSP70 silencing efficiency and evaluate downstream protein changes. HeLa cells were lysed in ice-cold RIPA buffer (Thermo Fisher Scientific, Cat. #89900) supplemented with a protease inhibitor cocktail (Roche, Cat. #11697498001), and lysates were incubated on ice for 20 min with periodic vortexing. Lysates were cleared by centrifugation at 14,000 × g for 15 min at 4 °C, and the supernatant was collected for protein analysis. Total protein concentration was determined using the Pierce BCA Protein Assay Kit (Thermo Fisher Scientific, Cat. #23227) according to the manufacturer’s instructions. An aliquot of 30 μg of protein was mixed with 4× Laemmli sample buffer, boiled for 5 min, and resolved on 10% SDS–polyacrylamide gels before being transferred onto PVDF membranes (Millipore, Cat. #IPVH00010) using a semi-dry transfer system. Membranes were blocked for 1 h at room temperature in 5% non-fat dry milk prepared in Tris-buffered saline containing 0.1% Tween-20 (TBST) and then incubated overnight at 4 °C with primary antibodies diluted in TBST containing 5% BSA. The following primary antibodies were used: anti-HSP70 (Cell Signaling Technology, 4872, 1:1,000), anti-G3BP1 (Abcam, ab56574, 1:1,000), and anti-GAPDH (Cell Signaling Technology, 5174, 1:5,000). After washing, membranes were incubated with HRP-conjugated anti-rabbit secondary antibody (Cell Signaling Technology, 7074, 1:3,000) for 1 h at room temperature. Signals were developed using an enhanced chemiluminescence (ECL) detection reagent (Thermo Fisher Scientific, Cat. #32106), and bands were visualized using the ChemiDoc imaging system (Bio-Rad). Densitometric quantifications of HSP70 and G3BP1 protein bands were made relative to the GAPDH loading control.

### 2.5 Flow cytometry

To visualize G3BP1-GFP fluorescence, cells were transfected with a G3BP1-GFP reporter plasmid (Addgene, Cat. #13521) 24 h prior to analysis. Transfection efficiency and reporter expression were confirmed by fluorescence microscopy. For the detection of misfolded and aggregated proteins, the ProteoStat® Aggresome Detection Kit (Enzo Life Sciences, Cat. #ENZ-51035) was used following the manufacturer’s protocol. In brief, cells were fixed, permeabilized, stained with the ProteoStat dye, and subsequently analyzed by flow cytometry to quantify intracellular protein aggregation. These combined assays enabled quantitative assessment of HSP70 knockdown-associated changes in protein homeostasis, G3BP1-GFP reporter fluorescence, and misfolded/aggregated protein accumulation. Flow cytometry was performed to quantify G3BP1-GFP fluorescence and misfolded protein accumulation following HSP70 knockdown. Cells stained with the ProteoStat® Aggresome Detection reagent or expressing the G3BP1-GFP reporter were harvested using gentle trypsinization, washed twice with ice-cold PBS, and resuspended in FACS buffer (PBS containing 2% FBS). Samples were analyzed on a BD FACSCanto II flow cytometer equipped with a 488 nm excitation laser; all samples were acquired using identical instrument settings and gated on single, live cells. Fluorescence signals were collected through the FITC detection channel. A minimum of 10,000 events per sample were acquired, and cell debris and doublets were excluded by standard forward-and side-scatter gating.

Data analysis was performed using FlowJo software version 10.10.0, and mean fluorescence intensity (MFI) was used as the quantitative metric for both ProteoStat® staining and G3BP1-GFP signal. Representative gating strategies are provided in Supplementary Figure S1. Cells were initially gated according to forward- and side-scatter characteristics to exclude debris. Doublets were removed using FSC-A *versus* FSC-H gating. MFI was calculated from the final singlet population. MFI was used as the quantitative metric for ProteoStat staining and total cellular G3BP1-GFP reporter fluorescence. Because flow cytometry measures total cellular fluorescence without providing spatial information, the G3BP1-GFP MFI measurement cannot establish intracellular puncta formation or stress granule assembly. Representative gating strategies are provided in Supplementary Figure S1, and representative histograms are shown in Supplementary Figures S2 and S3.

### 2.6 Statistical analysis

All experiments were performed using at least three independent biological replicates unless otherwise stated. Data are presented as the mean ± standard deviation (SD), with individual biological replicates displayed as scatter points where applicable. Statistical analyses were performed using Microsoft Excel. Comparisons between two groups were evaluated using an unpaired two-tailed Student’s t-test. Exact numbers of biological replicates are provided in the corresponding figure legends. Statistical significance was defined as *p < 0.05 and **p < 0.01.

## 3 Results

### 3.1 HSP70 knockdown significantly reduces transcript and protein abundance

To evaluate the efficiency of siRNA-mediated HSP70 knockdown, replicate HeLa cell cultures were transfected with Lipofectamine RNAiMAX and 50 nM siRNA. Cells were harvested 48 h after transfection for total RNA and protein isolation. RT-qPCR demonstrated that HSP70 siRNA reduced HSP70 mRNA abundance by approximately 92% relative to the non-targeting siRNA control (**p < 0.01; Figure 1A), indicating efficient HSP70 knockdown. Western blotting and densitometric analysis confirmed a >90% reduction in HSP70 protein abundance (Figures 1B,C), whereas total endogenous G3BP1 protein abundance remained unchanged (*p* > 0.1). These findings confirm efficient depletion of HSP70 at both the transcript and protein levels under the tested conditions.

**FIGURE 1:**
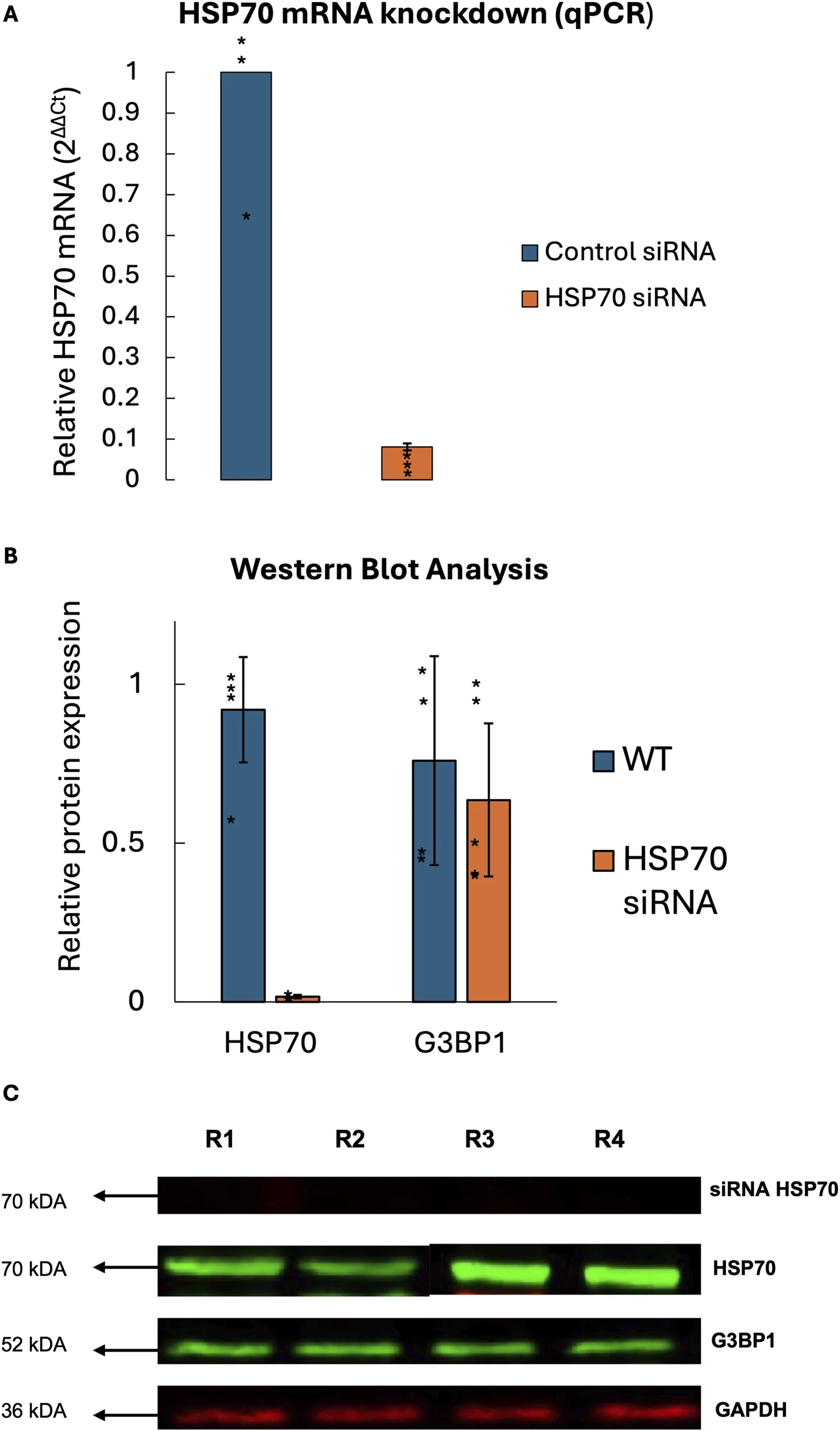
siRNA-mediated silencing suppresses HSP70 expression at the mRNA and protein levels in HeLa cells. **(A)** Relative HSP70 mRNA expression following HSP70 knockdown. Transcript levels were normalized to GAPDH and expressed relative to the control siRNA group, which was assigned a value of 1. Data are presented as the mean ± SD from three independent biological replicates (n = 3), and each dot represents one independent biological replicate. **(B)** HSP70 and G3BP1 protein abundance determined by Western blot densitometry and normalized to GAPDH. Data are presented as the mean ± SD from four independent biological replicates (n = 4), and each dot represents one independent biological replicate. **(C)** Representative immunoblot showing HSP70 (∼70 kDa), G3BP1 (∼52 kDa), and GAPDH (∼36 kDa) expression in control and HSP70-siRNA-treated cells. Statistical comparisons were performed using an unpaired two-tailed Student’s t-test; *p < 0.05 and **p < 0.01. Full-length uncropped immunoblots from all biological replicates, with molecular-weight markers visible, are provided in Supplementary Raw Blot File S1.

### 3.2 HSP70 depletion increases G3BP1-GFP fluorescence and ProteoStat signal

Flow-cytometric analysis showed that HSP70 depletion increased total cellular G3BP1-GFP MFI by 2.9-fold relative to the non-targeting siRNA control (**p < 0.01; Figure 2A). This measurement reflects total cellular reporter fluorescence and does not resolve G3BP1-GFP intracellular localization or puncta formation. It, therefore, does not directly demonstrate stress granule assembly. ProteoStat MFI increased by 1.8-fold following HSP70 depletion (Figure 2C), consistent with increased detection of intracellular misfolded or aggregated proteins.

**FIGURE 2:**
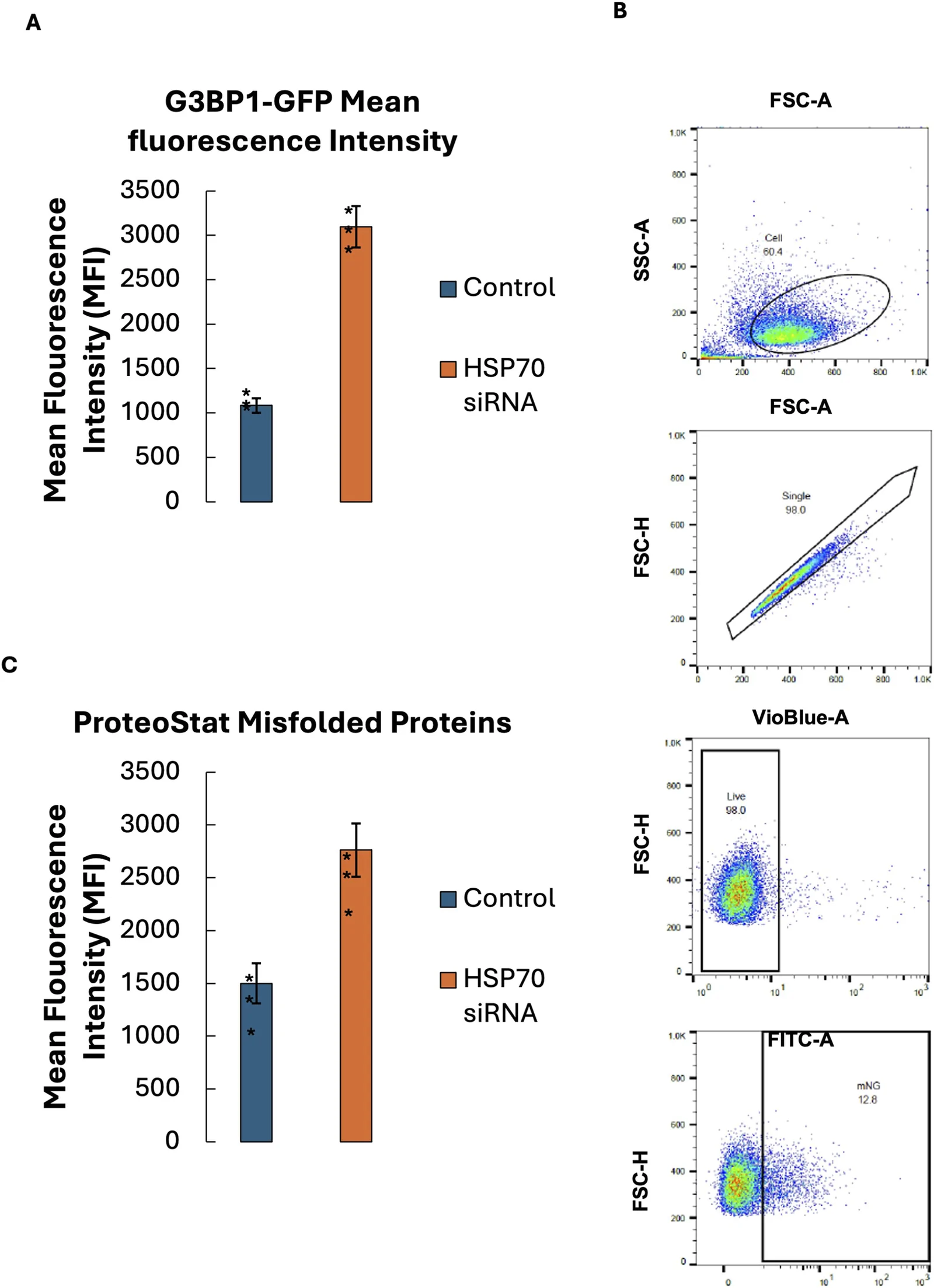
HSP70 knockdown increases total cellular G3BP1-GFP reporter fluorescence and ProteoStat-detected misfolded/aggregated protein burden in HeLa cells. **(A)** Quantification of total cellular G3BP1-GFP mean fluorescence intensity (MFI) by flow cytometry following HSP70 silencing. HSP70-depleted cells exhibited significantly increased G3BP1-GFP reporter fluorescence compared with control siRNA-transfected cells. Because flow cytometry does not resolve intracellular localization or puncta formation, this surrogate reporter signal does not constitute direct evidence of stress granule biogenesis. Data are presented as the mean ± SD from three independent biological replicates (n = 3), and each dot represents one independent biological replicate. **(B)** Representative flow-cytometry gating strategy showing sequential selection of intact cells and singlets before fluorescence quantification. **(C)** Quantification of intracellular misfolded/aggregated protein burden using ProteoStat staining. HSP70-depleted cells exhibited significantly increased ProteoStat fluorescence compared with control cells. Data are presented as the mean ± SD from three independent biological replicates (n = 3), and each dot represents one independent biological replicate. Statistical comparisons were performed using an unpaired two-tailed Student’s t-test; *p < 0.05 and **p < 0.01.

## 4 Discussion

Previous studies have shown that HSP70 contributes to proteostasis through its molecular chaperone activity (Ho et al., 2019; Bhattacharya and Picard, 2021; Deane and Brown, 2018). The present findings are consistent with this established role: acute siRNA-mediated HSP70 depletion in HeLa cells was associated with increased ProteoStat fluorescence and increased total cellular G3BP1-GFP reporter fluorescence. Importantly, the G3BP1-GFP measurement represents a surrogate reporter readout. It cannot distinguish increased reporter abundance from altered reporter solubility, intracellular redistribution, or incorporation into discrete puncta and therefore does not directly demonstrate stress granule assembly, phase separation, mRNP solubility changes, or physical interactions between HSP70 and RNA-binding proteins (Hu et al., 2022; Baymiller and Moon, 2023; Yuan et al., 2025). Immunofluorescence or confocal microscopy combined with quantitative analysis of G3BP1-positive puncta would be required to establish stress granule formation. A strength of the present study is its inhibitor-independent workflow, which combines transcript-level, protein-level, and flow-cytometric measurements under genetically defined conditions (Szewczyk et al., 2023; Jia et al., 2024). Nevertheless, the results should be interpreted as quantitative evidence of proteostasis-associated cellular responses following HSP70 depletion rather than as direct mechanistic evidence of stress granule biogenesis (Hu et al., 2022; Baymiller and Moon, 2023; Yuan et al., 2025).

The use of HeLa cells also places these findings within the broader context of cancer-associated proteostasis. Malignant cells frequently experience elevated proteotoxic stress arising from rapid proliferation, altered metabolism, genomic instability, and increased demands on protein synthesis and turnover. Molecular chaperones such as HSP70 can provide an adaptive advantage under these conditions by maintaining protein folding capacity and limiting the accumulation of damaged or misfolded proteins. Elevated HSP70 activity has consequently been associated with cancer cell survival and resistance to cellular stress and anticancer treatment. From this perspective, the increased ProteoStat signal following HSP70 depletion observed in the present study is consistent with reduced capacity to buffer proteotoxic burden. This raises the possibility that disruption of HSP70-dependent proteostasis may be particularly relevant in malignancies with a pronounced dependence on protein quality-control pathways. Such proteostasis dependence has been described in several aggressive malignancies, including multiple myeloma and subsets of breast cancer. Nevertheless, the present experiments were performed exclusively in HeLa cells and were not designed to evaluate therapeutic sensitivity; therefore, these broader cancer implications should be regarded as a rationale for future investigation rather than a therapeutic conclusion of the present study (Du et al., 2022; Ho et al., 2019; Bhattacharya and Picard, 2021; Deane and Brown, 2018; Baymiller and Moon, 2023; Yuan et al., 2025; Szewczyk et al., 2023; Jia et al., 2024).

Proteostasis is also closely interconnected with cellular redox homeostasis. Reactive oxygen species can promote oxidative modification, destabilization, and misfolding of proteins, thereby increasing the demand placed on molecular chaperone and protein degradation systems. HSP70 has been implicated in cellular adaptation to oxidative stress and protection against ROS-associated protein damage. Thus, although the increased ProteoStat signal observed following HSP70 depletion primarily indicates a greater burden of misfolded or aggregated proteins, an interaction with altered redox homeostasis cannot be excluded. Importantly, ROS production, antioxidant capacity, and redox-sensitive signaling were not measured in the present study, and the observed phenotype therefore cannot be attributed specifically to redox dysregulation.

HSP70-associated proteostasis has potential disease relevance across several settings, although the present study does not evaluate disease causation or therapeutic efficacy. In cancer, HSP70 and associated stress-response pathways can support cellular survival under proteotoxic and endoplasmic reticulum stress, suggesting that modulation of chaperone activity could be investigated as part of future combination-treatment strategies (Mesci et al., 2025). Stress granule pathways are likewise being explored as pharmacological targets, including through natural products and G3BP-associated mechanisms (Fan et al., 2025). More broadly, HSP70-related stress adaptation has been associated with inflammatory and heat-stress responses (Greene et al., 2024), whereas altered protein-quality control and oxidative-stress mechanisms contribute to neurotoxicity and neurodegenerative disease processes (Jahani and Mohammadi, 2025). Disease-specific therapeutic approaches must nevertheless remain context dependent, as illustrated by emerging targeted treatments in vitiligo (Dan et al., 2025) and strategies intended to enhance immunogenic cell death in cancer (He et al., 2024). These studies provide broader translational context, but the present HeLa-cell findings cannot establish clinical efficacy, therapeutic safety, or applicability to other diseases.

This relationship may also be relevant to cancer stem cell biology. Cancer stem-like cells rely on coordinated proteostasis, metabolic adaptation, and redox-regulatory mechanisms to maintain survival under adverse microenvironmental and therapeutic conditions. Redox-sensitive regulators, including NRF2, BACH1, and HIF-1α, have been implicated in cancer stem cell maintenance, metabolic reprogramming, epithelial–mesenchymal plasticity, and therapeutic resistance. In this context, investigating how HSP70-dependent proteostasis intersects with redox signaling in cancer stem-like populations represents an interesting extension of the present workflow. However, cancer stem-cell phenotypes and these signaling pathways were not examined in the present study; therefore, this connection should be regarded solely as a direction for future investigation rather than as a mechanistic conclusion (Du et al., 2024).

Several limitations should be acknowledged. First, all experiments were conducted exclusively in HeLa cells. Although HeLa cells provide an established model for investigating molecular chaperone biology, validation in additional cancer models, non-transformed cells, and disease-relevant cell types is required before the biological findings or broader applicability of the workflow can be generalized. Second, the experiments were performed under standard culture conditions without pharmacological or severe external stress induction. Although this approach reduces confounding by strong exogenous stressors, it limits generalizability to proteotoxic, oxidative, or thermal stress conditions under which HSP70 activity may be particularly important.

Third, only one HSP70-targeting siRNA duplex was evaluated. Consequently, sequence-dependent off-target effects cannot be excluded, and the observed phenotypes cannot yet be attributed unequivocally to HSP70 loss. Validation using a second independent HSP70-targeting siRNA duplex, followed by confirmation of knockdown efficiency and repetition of the ProteoStat and G3BP1-GFP measurements, will be required to establish on-target reproducibility. Fourth, rescue experiments restoring HSP70 expression were not performed; therefore, a direct causal relationship between HSP70 depletion and the observed phenotypes remains to be established.

Fifth, potential compensatory induction of other HSP-family chaperones, including HSPA1A and HSPA8, was not assessed. Such compensatory responses could attenuate or otherwise modify the magnitude of the proteostasis defects observed following acute HSP70 depletion. Future studies should therefore assess major HSP paralogs at transcript and protein levels. Sixth, only a single post-transfection time point of 48 h was analyzed. The present dataset consequently cannot resolve early *versus* late responses, progressive accumulation of proteostasis defects, or adaptive compensation over time. Time-course studies will be required to characterize the temporal sequence of responses following HSP70 depletion.

Finally, flow-cytometric measurement of G3BP1-GFP MFI lacks spatial resolution and represents only a surrogate reporter signal. It does not constitute direct evidence of stress granule biogenesis and cannot determine granule number, size, formation, or intracellular localization. Immunofluorescence or confocal microscopy combined with quantitative analysis of G3BP1-positive puncta will be required to directly evaluate stress granule assembly (Yang et al., 2020; Smith and Bartel, 2025; Hu et al., 2022).

## 5 Conclusion

siRNA-mediated HSP70 knockdown reduced HSP70 transcript and protein levels by >90% and was accompanied by an approximately 1.8-fold increase in ProteoStat-detected misfolded/aggregated protein burden and an approximately 2.9-fold increase in total cellular G3BP1-GFP reporter fluorescence. The G3BP1-GFP result represents a surrogate reporter response and does not directly demonstrate stress granule assembly. Collectively, the findings support the utility of this inhibitor-independent workflow for assessing proteostasis-associated cellular responses following acute HSP70 depletion. Confirmation using a second independent siRNA duplex, microscopy-based puncta analysis, rescue experiments, compensatory chaperone profiling, additional cellular models, and temporal analyses will be required to establish target specificity, broader applicability, and underlying mechanisms (Sui et al., 2020; Frydrýšková et al., 2020; Zhang et al., 2022; Brenner et al., 2023; Chierichetti et al., 2023; Cristofani et al., 2021; Sciandrone et al., 2023).

## Data Availability

The original contributions presented in the study are included in the article/Supplementary Material, further inquiries can be directed to the corresponding author/s.
