# Supplementary material for: Quantitative analysis of proteostasis disruption following siRNA-mediated HSP70 depletion in HeLa cells
Source: Front Genet. 2026 Sep 28;17:1856671. doi: 10.3389/fgene.2026.1856671 (PMC13654591; doi:10.3389/fgene.2026.1856671)
Supplement: Supplementary file 1 [file DataSheet1.pdf]

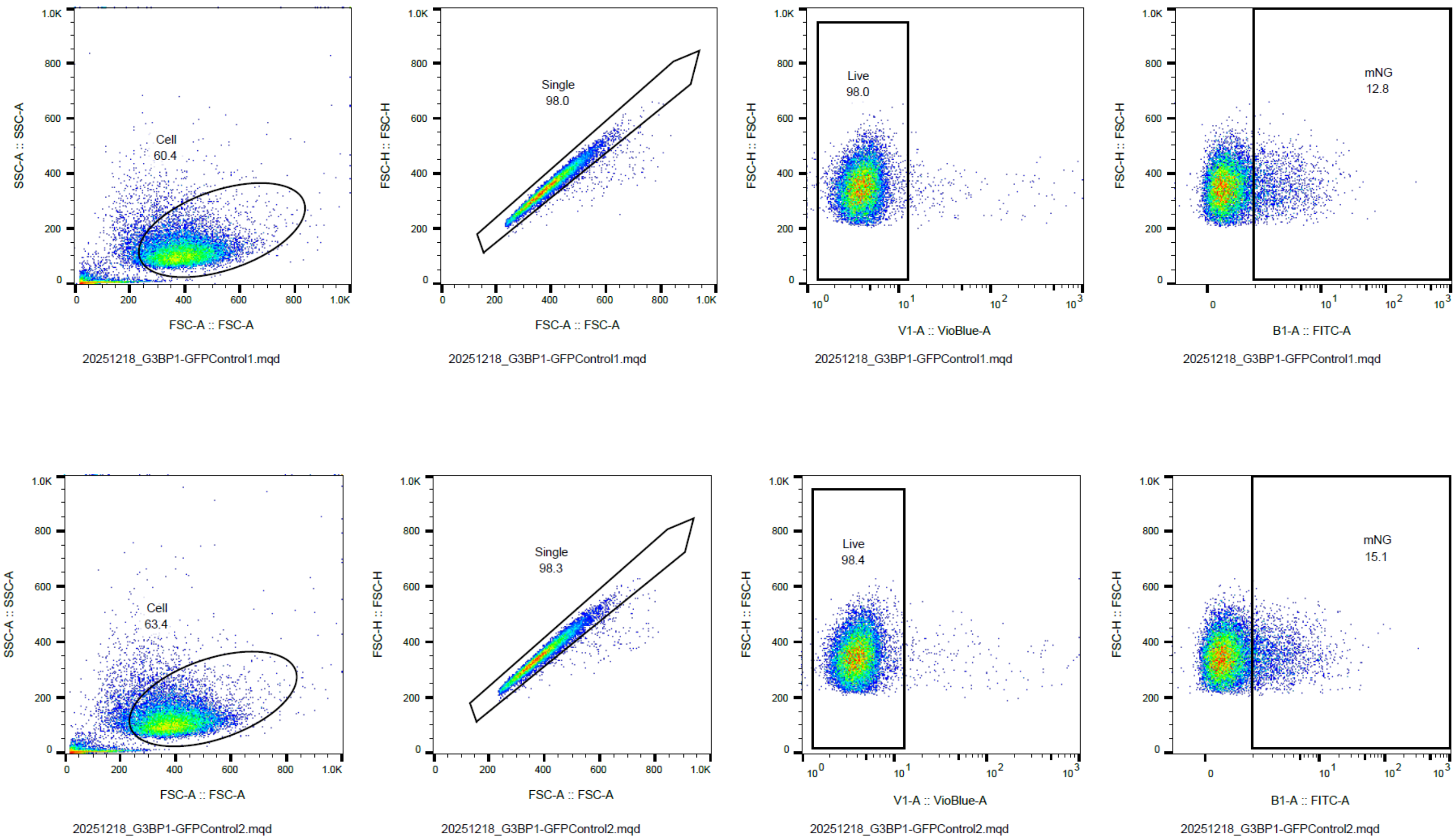

Cell
60.4
SSC-A :: SSC-A
FSC-A :: FSC-A
20251218_G3BP1-GFPControl1.mqd
Single
98.0
FSC-H :: FSC-H
FSC-A :: FSC-A
20251218_G3BP1-GFPControl1.mqd
Live
98.0
FSC-H :: FSC-H
V1-A :: VioBlue-A
20251218_G3BP1-GFPControl1.mqd
mNG
12.8
FSC-H :: FSC-H
B1-A :: FITC-A
20251218_G3BP1-GFPControl1.mqd
Cell
63.4
SSC-A :: SSC-A
FSC-A :: FSC-A
20251218_G3BP1-GFPControl2.mqd
Single
98.3
FSC-H :: FSC-H
FSC-A :: FSC-A
20251218_G3BP1-GFPControl2.mqd
Live
98.4
FSC-H :: FSC-H
V1-A :: VioBlue-A
20251218_G3BP1-GFPControl2.mqd
mNG
15.1
FSC-H :: FSC-H
B1-A :: FITC-A
20251218_G3BP1-GFPControl2.mqd

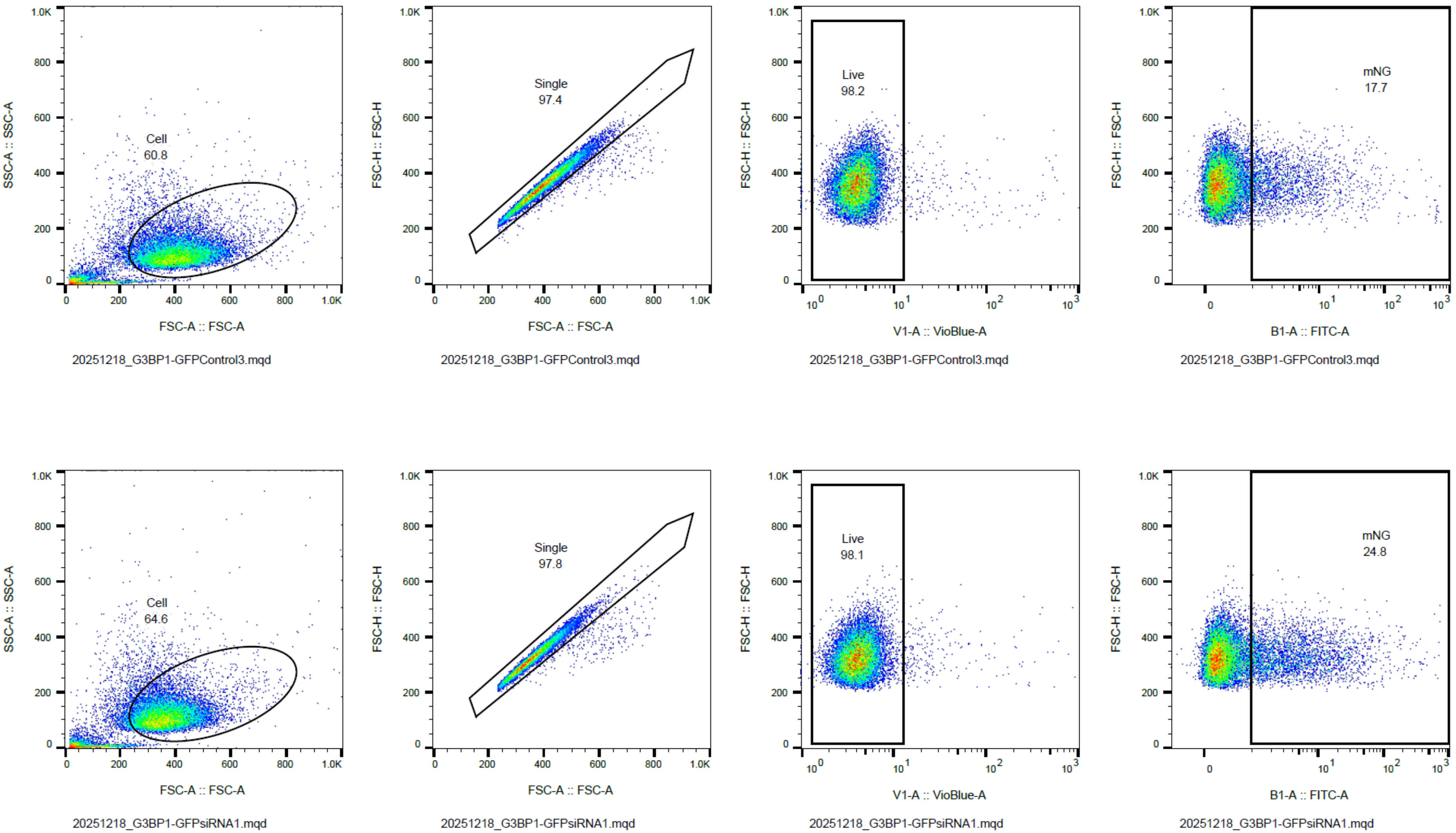

Cell
60.8
SSC-A :: SSC-A
FSC-A :: FSC-A
20251218_G3BP1-GFPControl3.mqd
Single
97.4
FSC-H :: FSC-H
FSC-A :: FSC-A
20251218_G3BP1-GFPControl3.mqd
Live
98.2
FSC-H :: FSC-H
V1-A :: VioBlue-A
20251218_G3BP1-GFPControl3.mqd
mNG
17.7
FSC-H :: FSC-H
B1-A :: FITC-A
20251218_G3BP1-GFPControl3.mqd
Cell
64.6
SSC-A :: SSC-A
FSC-A :: FSC-A
20251218_G3BP1-GFPsiRNA1.mqd
Single
97.8
FSC-H :: FSC-H
FSC-A :: FSC-A
20251218_G3BP1-GFPsiRNA1.mqd
Live
98.1
FSC-H :: FSC-H
V1-A :: VioBlue-A
20251218_G3BP1-GFPsiRNA1.mqd
mNG
24.8
FSC-H :: FSC-H
B1-A :: FITC-A
20251218_G3BP1-GFPsiRNA1.mqd

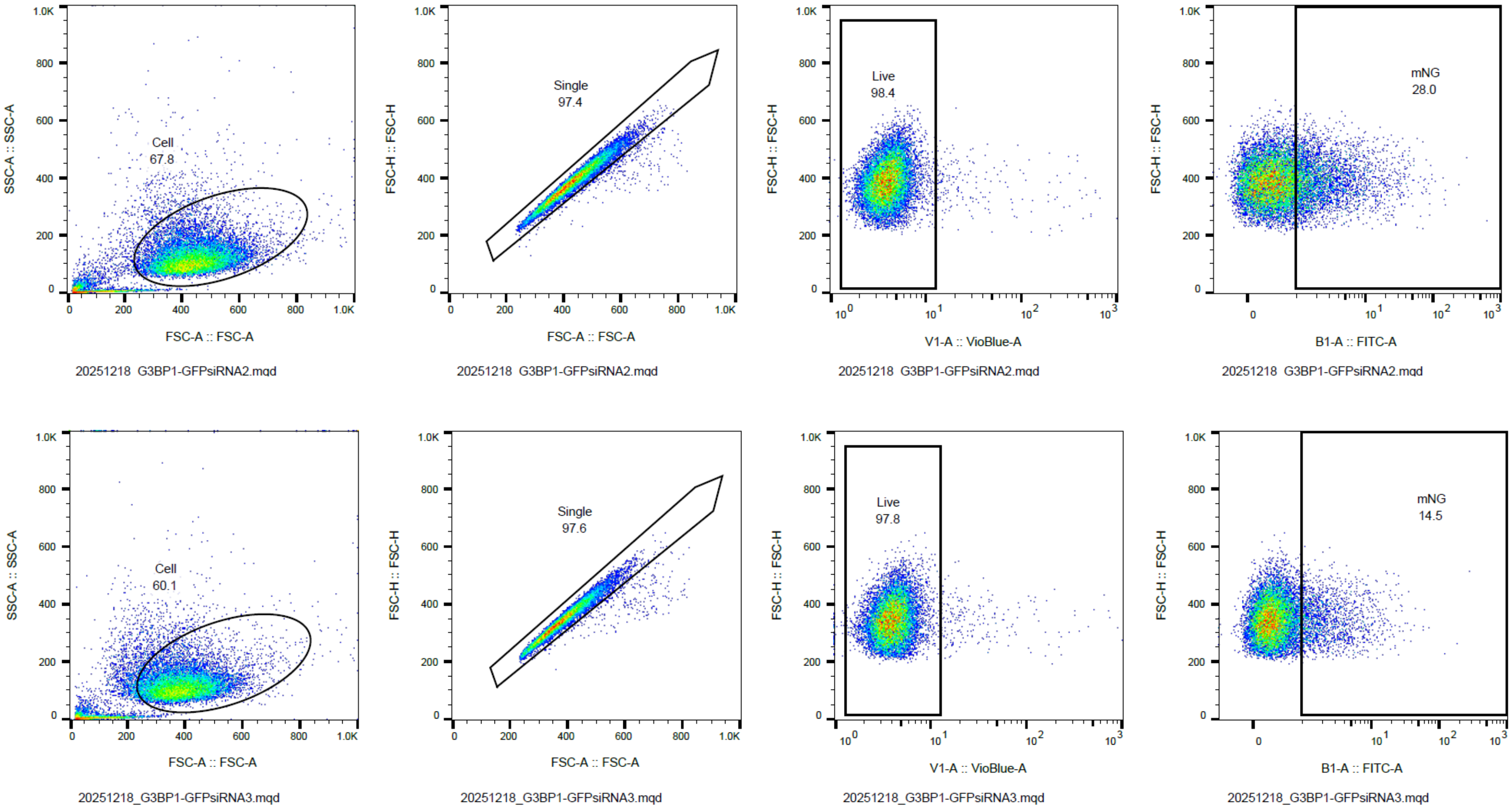
Cell
67.8
SSC-A :: SSC-A
FSC-A :: FSC-A
20251218_G3BP1-GFPsiRNA2.mqd
Single
97.4
FSC-H :: FSC-H
FSC-A :: FSC-A
20251218_G3BP1-GFPsiRNA2.mqd
Live
98.4
FSC-H :: FSC-H
V1-A :: VioBlue-A
20251218_G3BP1-GFPsiRNA2.mqd
mNG
28.0
FSC-H :: FSC-H
B1-A :: FITC-A
20251218_G3BP1-GFPsiRNA2.mqd
Cell
60.1
SSC-A :: SSC-A
FSC-A :: FSC-A
20251218_G3BP1-GFPsiRNA3.mqd
Single
97.6
FSC-H :: FSC-H
FSC-A :: FSC-A
20251218_G3BP1-GFPsiRNA3.mqd
Live
97.8
FSC-H :: FSC-H
V1-A :: VioBlue-A
20251218_G3BP1-GFPsiRNA3.mqd
mNG
14.5
FSC-H :: FSC-H
B1-A :: FITC-A
20251218_G3BP1-GFPsiRNA3.mqd

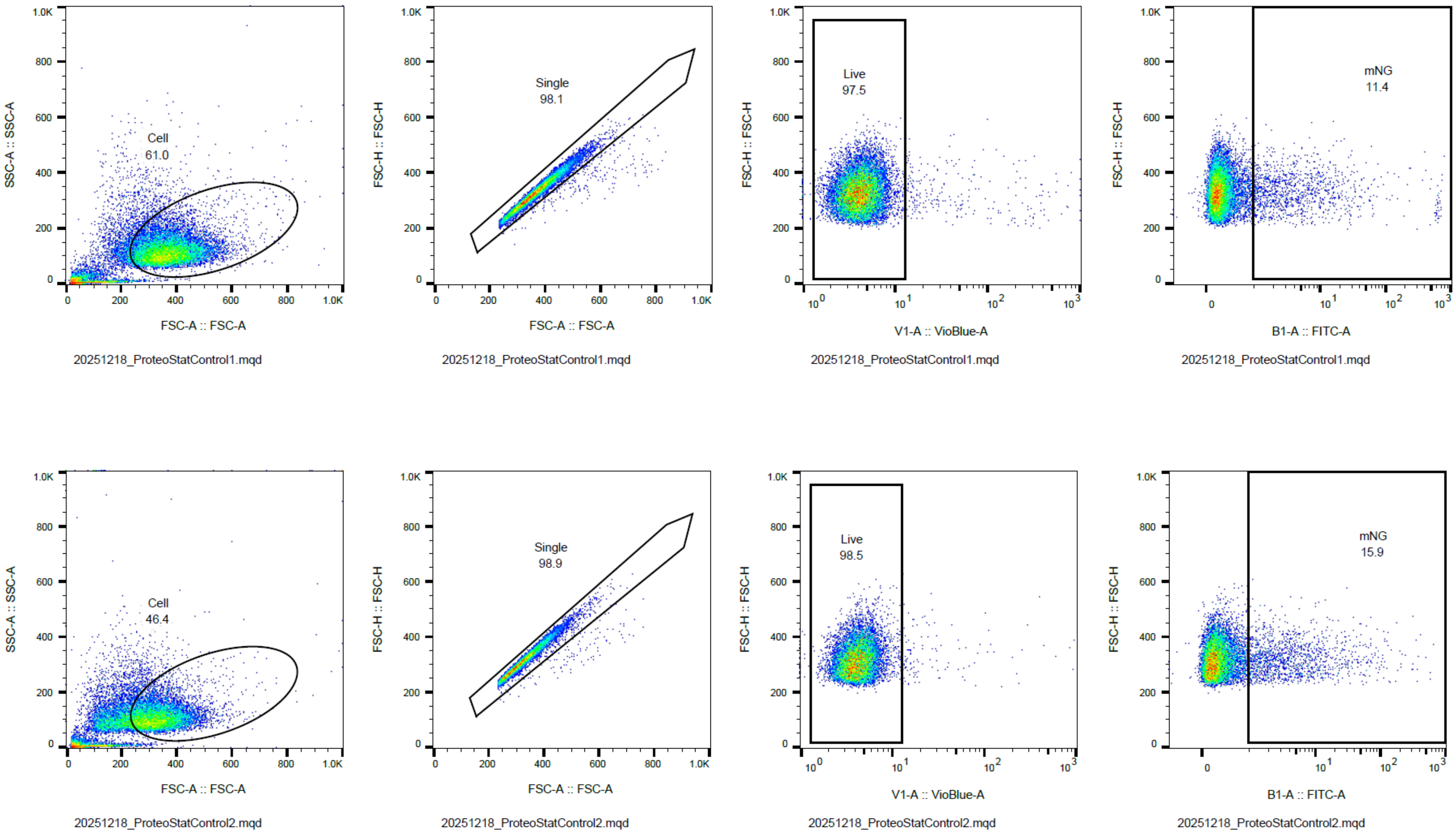
Cell
61.0
SSC-A :: SSC-A
FSC-A :: FSC-A
20251218_ProteoStatControl1.mqd
Single
98.1
FSC-H :: FSC-H
FSC-A :: FSC-A
20251218_ProteoStatControl1.mqd
Live
97.5
FSC-H :: FSC-H
V1-A :: VioBlue-A
20251218_ProteoStatControl1.mqd
mNG
11.4
FSC-H :: FSC-H
B1-A :: FITC-A
20251218_ProteoStatControl1.mqd
Cell
46.4
SSC-A :: SSC-A
FSC-A :: FSC-A
20251218_ProteoStatControl2.mqd
Single
98.9
FSC-H :: FSC-H
FSC-A :: FSC-A
20251218_ProteoStatControl2.mqd
Live
98.5
FSC-H :: FSC-H
V1-A :: VioBlue-A
20251218_ProteoStatControl2.mqd
mNG
15.9
FSC-H :: FSC-H
B1-A :: FITC-A
20251218_ProteoStatControl2.mqd

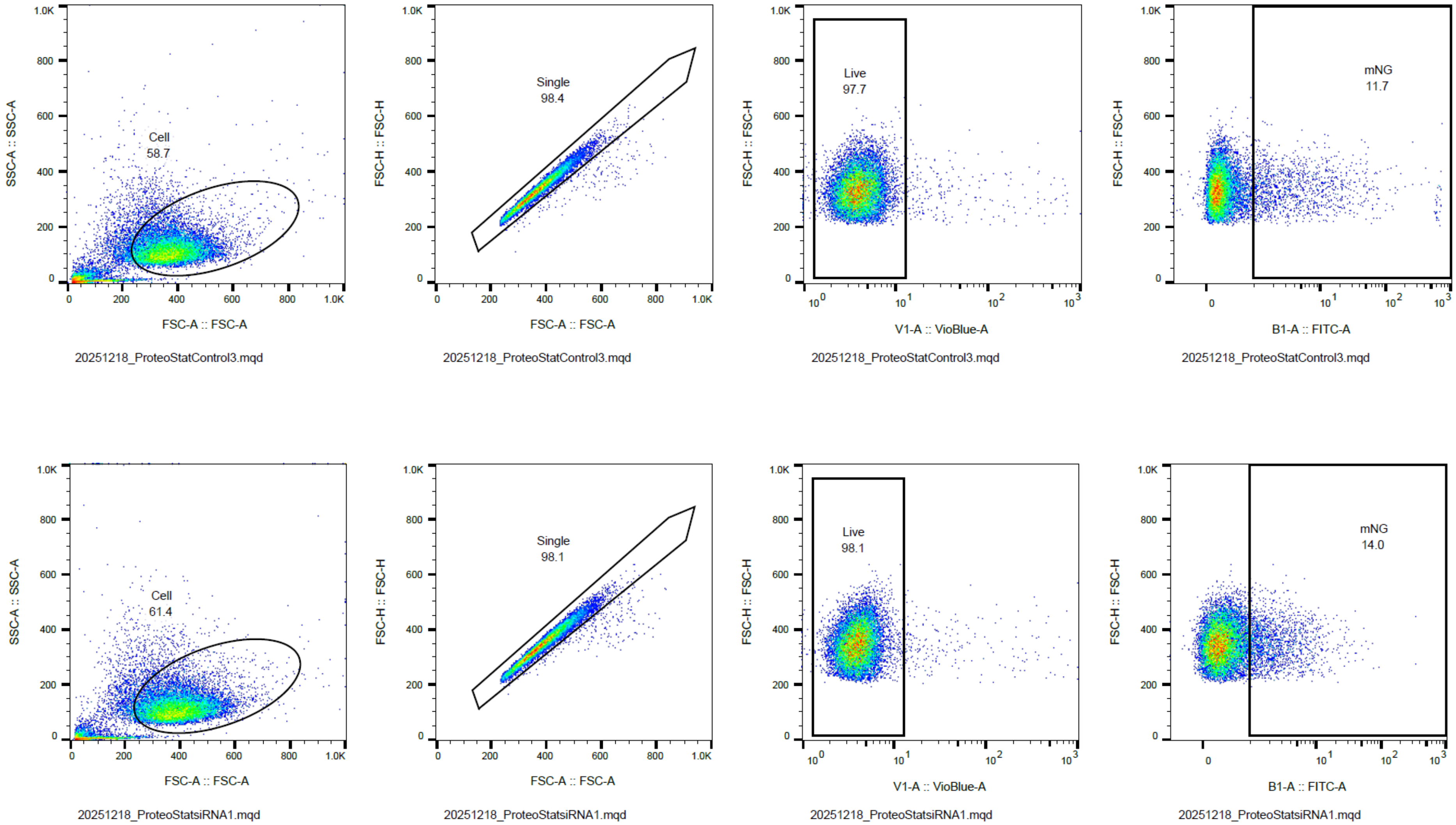
Cell
58.7
SSC-A :: SSC-A
FSC-A :: FSC-A
20251218_ProteoStatControl3.mqd
Single
98.4
FSC-H :: FSC-H
FSC-A :: FSC-A
20251218_ProteoStatControl3.mqd
Live
97.7
FSC-H :: FSC-H
V1-A :: VioBlue-A
20251218_ProteoStatControl3.mqd
mNG
11.7
FSC-H :: FSC-H
B1-A :: FITC-A
20251218_ProteoStatControl3.mqd
Cell
61.4
SSC-A :: SSC-A
FSC-A :: FSC-A
20251218_ProteoStatsiRNA1.mqd
Single
98.1
FSC-H :: FSC-H
FSC-A :: FSC-A
20251218_ProteoStatsiRNA1.mqd
Live
98.1
FSC-H :: FSC-H
V1-A :: VioBlue-A
20251218_ProteoStatsiRNA1.mqd
mNG
14.0
FSC-H :: FSC-H
B1-A :: FITC-A
20251218_ProteoStatsiRNA1.mqd

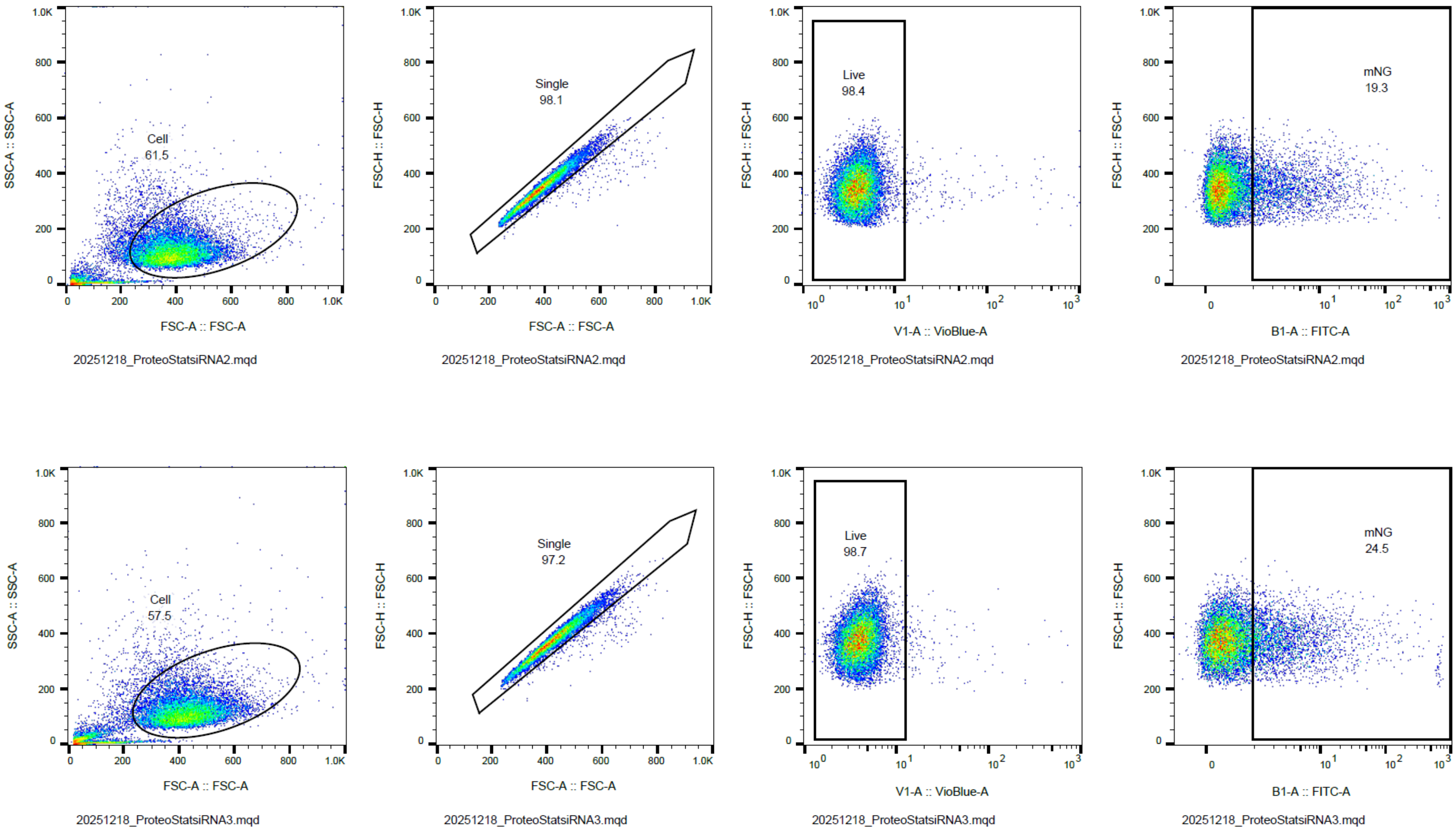
Cell
61.5
SSC-A :: SSC-A
FSC-A :: FSC-A
20251218_ProteoStatsiRNA2.mqd
Single
98.1
FSC-H :: FSC-H
FSC-A :: FSC-A
20251218_ProteoStatsiRNA2.mqd
Live
98.4
FSC-H :: FSC-H
V1-A :: VioBlue-A
20251218_ProteoStatsiRNA2.mqd
mNG
19.3
FSC-H :: FSC-H
B1-A :: FITC-A
20251218_ProteoStatsiRNA2.mqd
Cell
57.5
SSC-A :: SSC-A
FSC-A :: FSC-A
20251218_ProteoStatsiRNA3.mqd
Single
97.2
FSC-H :: FSC-H
FSC-A :: FSC-A
20251218_ProteoStatsiRNA3.mqd
Live
98.7
FSC-H :: FSC-H
V1-A :: VioBlue-A
20251218_ProteoStatsiRNA3.mqd
mNG
24.5
FSC-H :: FSC-H
B1-A :: FITC-A
20251218_ProteoStatsiRNA3.mqd
