# Supplementary material for: Quantitative analysis of proteostasis disruption following siRNA-mediated HSP70 depletion in HeLa cells
Source: Front Genet. 2026 Sep 28;17:1856671. doi: 10.3389/fgene.2026.1856671 (PMC13654591; doi:10.3389/fgene.2026.1856671)
Supplement: Supplementary file 3 [file Table3.docx]

Uncropped western blot: Replicas

20

25

37

50

75

100

Control r4

Control r3

siRNA r4

siRNA r5

GAPDH 36 KDA

HSP70 70 KDA
